# Root and Shoot Growth of a Modern and an Old Tall Durum Wheat (*Triticum durum* Desf.) Variety under Dual-Purpose Management

**DOI:** 10.3390/plants12030588

**Published:** 2023-01-29

**Authors:** Roberta Rossi, Giovanni Bitella, Rocco Bochicchio, Rosanna Labella, Francesco Angerame, Marcella Urbano, Mariana Amato

**Affiliations:** 1 Council for Agricultural Research and Economics, Research Centre for Animal Production and Aquaculture (CREA-ZA), 85051 Bella-Muro (Potenza), Italy; 2 School of Agriculture, Forestry, Food and Environmental Sciences, University of Basilicata, 85100 Potenza, Italy; 3 Institute of Biosciences and Bioresources, CNR (National Research Council), 70126 Bari, Italy

**Keywords:** dual-purpose wheat, ancient tall variety, root length

## Abstract

In dual-purpose cereal systems, the co-production of fodder and grain can increase farm profitability and reduce farming risks. Our work evaluated shoot and root growth in durum wheat (*Triticum durum* Desf.) under dual-purpose management in a medium-high rainfall area of southern Italy. We compared a modern variety (Core) with a tall ancient variety (Saragolle lucana) under traditional (NDP) and dual-purpose (DP) management and tested the hypothesis that clipping plants during the vegetative stage would reduce root growth and dewatering before anthesis, which is advantageous in drought-prone environments. Experiments were conducted in Bella (PZ), Basilicata region, southern Italy (40°42′ N, 15°32′ E) on a clay loam soil in 2021 in a split-plot design on 2 × 2 main plots and 1 × 2 split-plots with 6 replicates. The DP treatment consisted of simulated grazing by clipping plants at 5 cm from the ground 3 months after sowing (at first hollow stem). Forage Biomass was not different at *p* = 0.05 between varieties, with an average of 0.58 t ha^−1^ DM. Grain yield was not penalized by clipping (*p* = 0.05) and did not differ significantly between varieties. SPAD was always lower in the Saragolle variety and lowered by clipping. Defoliation delayed phenology in both cultivars but did not reduce the final number of spikes per square meter. Stomatal conductance was correlated to temperature, did not differ between cultivars, and was not influenced by clipping. Soil water depletion was monitored in modern wheat from the booting stage to the beginning of grain filling. Clipping did not result in a reduction in pre-anthesis water depletion, possibly due to evaporative losses. Root density was markedly reduced by clipping in core variety between 0.20 and 0.60 m and much less in Saragolle. Unclipped Saragolle produced thicker roots and higher root masses compared to clipped plants. Defoliated Saragolle shifted to finer roots, reducing root mass more than length. This may have reduced the metabolic cost of soil exploration, thereby increasing root foraging efficiency.

## 1. Introduction

In Mediterranean countries, cereal production and animal husbandry were traditionally integrated through herbage and stubble grazing [1]. Nowadays, though, the large majority of cereal enterprises are specialized, and despite the direct and indirect benefits of land sharing, cereal grazing has become rare and the agronomic know-how has faded. In Australia, to promote the adoption of dual-purpose systems, a national research program was launched (“Grain and Graze”), with the aim of studying facilitative interactions and defining crop and grazing management protocols [2]. By definition, dual-purpose cereal systems have a double goal: the production of grain and the co-production of forage during the winter. The two uses are possible by applying appropriate grazing and agronomic strategies to reduce grain yield penalties. Under appropriate management, dual-purpose systems can increase net income [3,4] and reduce farming risks by diversifying the sources of income [5]. The most obvious return is for the livestock sector: winter cereal grazing provides an extra quality-forage source during the so-called winter “feeding gap” [6]. Wheat vegetative biomass is characterized by a high protein content (22.38%) and in vitro digestibility (83.43%), which has been shown to allow rapid weight gain in grazing lambs [7]. In northern Australia, when dual-purpose crops were used as the primary weaning feed source, ewe’s yearlings’ sale weight increased by 16% [5]. Grazing wheat has the indirect benefit of sparing natural pastures during winter to capitalize on spring grazing. Dove et al. [8] showed that in favorable years, grazing wheat tripled total forage production compared to grazing only (2.5–3 t DM ha^−1^ year^−1^ vs. 1 t DM ha^−1^ year^−1^). A simulation study showed that in areas with high annual rainfall availability, adopting dual-purpose wheat and rapeseed cultivation on a small percentage of the farm area (10.20%) increased grazing days by 10–15% and increased production by 25% compared to grazing only [3]. For wheat enterprises, dual-purpose management generally reduces grain yield (on average 7%, with wide variations due to soil and climate). However, this penalty is compensated for by a series of benefits such as the reduction in lodging and residue build-up (which facilitates machinery and reduces pathogenic fungi burden). By delaying phenology, clipping can reduce the risk of late frost damage [2]. There are also advantages related to farm risk mitigation: multipurpose for crop use can contribute to reducing the impact of grain price oscillations or compensate yield losses due to climate variability [9]. Substantial research has been dedicated to the effect of clipping on yield in order to define the optimal grazing windows and intensity [10,11,12,13,14]. Dual-purpose management requires specific adaptations such as grazing tolerance. Cultivars also need to display biotic stress tolerance: early sowing (which increases forage yield potential) can be associated with a greater incidence of root rot disease [15]. Giunta et al. [16] defined desirable phenology and related agronomic traits for adaptation to dual-purpose exploitation in winter cereals (triticale, barley, and durum wheat). In Australia, grain companies already supply many cultivars specifically suitable for dual-purpose systems (e.g., AusWest & Stephen Pasture Seeds; Australian Grain Technologies). A few choices have also been made available for the Mediterranean from Italian seed companies (e.g., adagliosementi.com and Padana sementi). In many regions, water availability, especially around the drought-sensitive stage of anthesis, is the most important yield-limiting factor. Dual-purpose management has been envisaged as a possible water-saving agronomic practice. By reducing leaf area, clipping also reduces transpiration and slows down water use after clipping. This deferred water use can result in extra water being available during the drought-sensitive stages of anthesis and grain filling. The value of post-anthesis soil water reserve was quantified for wheat by [17]: under moderate drought stress, 10.5 mm of additional water left in the soil after anthesis increased wheat yield by 0.62 t/ha; water use efficiency during this stage is at its maximum as every drop of water used is converted into grain. Virgona et al. [18] found that under dual-purpose defoliation, wheat water consumption was reduced soon after grazing, and this water was used later during grain filling, thereby increasing crop water use efficiency. Clipping effects on water use efficiency are weather dependent: in years characterized by a prolonged drought after clipping, delayed water consumption increases yield (if this water is not lost by evaporation). In wet years, however, defoliation caused by reducing plant uptake capacity can leave substantial water unused and hence determine an inefficient use of resources [19]. Besides the reduction in transpiring leaf area, clipping can also reduce or delay root growth and thus defer water uptake rates until roots recover enough density. Early research on prairie grasses showed that defoliation slows root downward growth, lateral spread, and total density as a function of grazing intensity [20]. Complete defoliation slowed down root extension within a few hours in native grasses and lucerne [21]. Paez Garcia et al. [22] found that 40% spring wheat shoot removal during tillering stage reduced root mass from 35% to 55%, while complete shoot removal reduced root density by 83% for the grazing-sensitive cultivar Cheyenne and by 68% for the grazing tolerant cultivar Duster. Defoliation reduced wheat root proliferation within 1 h after clipping, and this is probably the result of a decrease in carbon supply; it also caused a quick decrease in soluble sugars in the apical part of the growth zone (Bingham et al., 1996). Regarding wheat root growth under dual purpose [23], it was shown that root depth was unaffected by defoliation and no effect was found on root mass unless defoliation occurred very early (4 leaf stage) and repeatedly, so that primary root deepening was arrested and nodal root growth reduced. The rooting pattern shows substantial variation among genotypes, and there is an active research front on root phenotyping for the selection of useful root traits [24,25]. Root ideotypes for dual-purpose systems have not been yet defined. There is a growing body of literature on dual-purpose systems in Italy, including research on the suitability of ancient tall varieties [16,26,27], and so far, this research has been mostly focused on phenology; no data are yet available on water use patterns. There is literature indicating that ancient wheat cultivars invest more in roots compared to modern varieties [28,29], but to the best of our knowledge, the effect of clipping on root growth has never been tested on ancient wheat cultivars. The objective of our work was to compare a modern durum wheat and an ancient tall variety under dual purpose. We tested the null hypothesis that early crop defoliation during the vegetative stage and wheat variety would not affect durum wheat phenology, above- and below-ground biometry, yield, and plant-soil characteristics relevant to water relations. A specific target of our research was root behavior under dual-purpose; we, therefore, set the null hypotheses that defoliated and intact plants and the tested ancient and modern wheat varieties did not exhibit different root mass, length, diameter, or derived indicators. We also tested interactions through the null hypothesis that responses of varieties to defoliation were not different.

## 2. Results

### 2.1. Fodder, Grain Yield and Weather

Wheat was clipped at the end of the tillering stage before the crops developed the first hollow stem. Late sowing (15 November) two weeks later than the usual sowing dates for the area narrowed the forage growth window to just 70 days (clipping was performed on 15 February 2021), and at the time of clipping, dry forage yield was only 0.58 t/ha on average with no significant difference between varieties (*p*-value > 0.05). Fresh weight corresponded to 2.97 and 2.90 t/ha, respectively, for Core and Saragolle with no significant differences. As a measure of post-grazing recovering capacity, biomass was measured again 2.5 months after cutting during the reproductive stage. Both cultivars recovered aboveground biomass after cutting, and no significant differences between clipped and unclipped plots were found for both Core (2.387 vs. 2.811 DM t ha^−1^ n.s.) and Saragolle (2.31 vs. 2.314 t ha^−1^ n.s.). Data on grain yield are reported in Table 1. 

Yield was not penalized by clipping, showing relatively small, non-significant differences between clipped and unclipped plots (−8% for Core and +2% for Saragolla, *p*-value > 0.05). The interaction between treatment and variety was not significant, but the yield advantage of Core over Saragolle was larger for unclipped plots (+31%) than for clipped plots (+22%).

**Figure 1:**
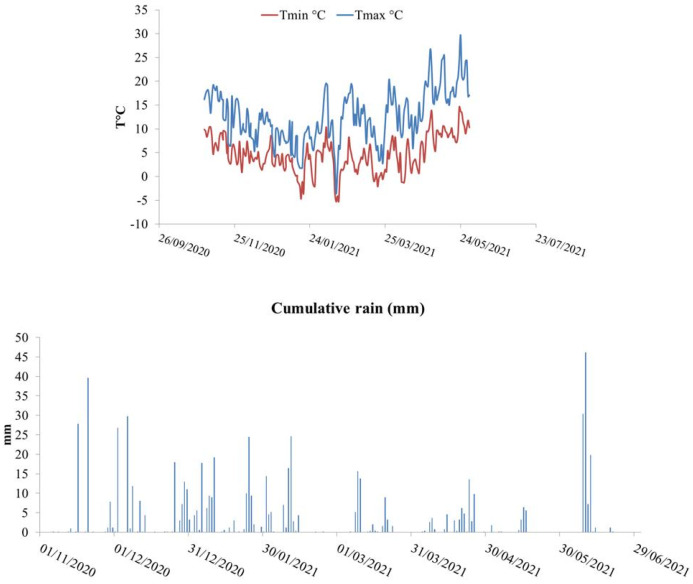
Meteorological data: daily minimum (Tmin °C) and maximum (Tmax °C) (**top**) and cumulative rain during the growth season 2020–2021 (**bottom**).

Meteorological data are reported in Figure 1. From sowing to harvest, it rained 687 mm, and precipitation was concentrated in winter months (73% of total rain).

From sowing to anthesis, the crop received 580 mm of water. After clipping, there was a dry spell of 20 days. From clipping to anthesis (about 95 days later), the crop received an additional 129 mm of rainwater in the form of sparse rain showers (39 rain events); rain volume ranged from 0.2 mm to 15.6, with an average of 3.5 mm per rain event. During grain filling, the crop received an extra 106 mm of rainwater that concentrated soon after anthesis. Regarding temperatures, the lowest temperature was recorded in February, and our winter minimum temperatures were around 1 °C > than the average. The mean value of the maximum temperature around anthesis (May) matched the long-term average (20 °C).

### 2.2. Phenology and SPAD Index

**Figure 2:**
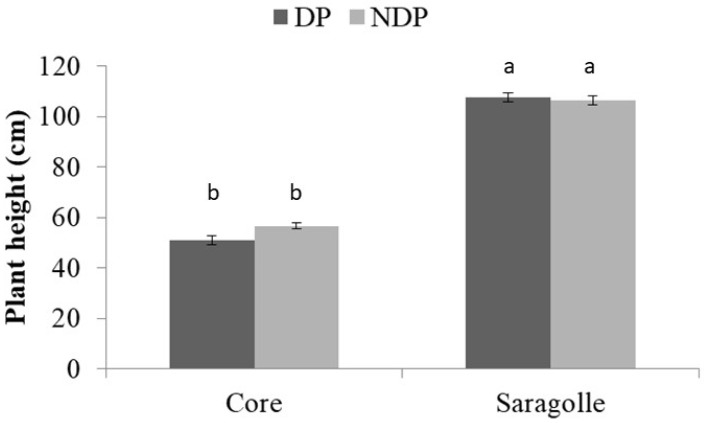
Interaction of wheat variety and clipping management on plant height on 20 May 2021. Bar plots of the mean values and relative standard error bars. Different lowercase letters above the bars indicate significant differences at *p* < 0.05.

There was a significant difference in phenology between cultivars: Saragolle was almost 2 weeks behind Core, and clipping delayed phenology in both cultivars. During the first week of May (4 May), the Core variety had reached the heading stage with advanced phenology in unclipped plots (150 vs 248 emerged spikes m^−2^ for clipped vs unclipped plants, respectively) (*p*-value < 0.05), while Saragolle was still in the booting stage, and no spikes had emerged in both clipped and unclipped plots. By 13 May, the Core variety had reached anthesis, showing a small, non-significant difference in spike density between treatments (450 and 500 spikes m^−2^ in clipped and unclipped plots, respectively). By that date, the Saragolle variety was still behind (heading stage) but similarly delayed by clipping, with 93 vs 185 emerged spikes m^−2^ in clipped vs unclipped plots, respectively, *p*-value < 0.05). By 20 Ma, the Saragolle variety had reached the flowering stage, while grain filling had begun for Core. Plant height (measured from the base of the stem to the last node) of the Saragolle variety was twice that of the Core variety (Figure 2) but was not affected by clipping.

**Figure 3:**
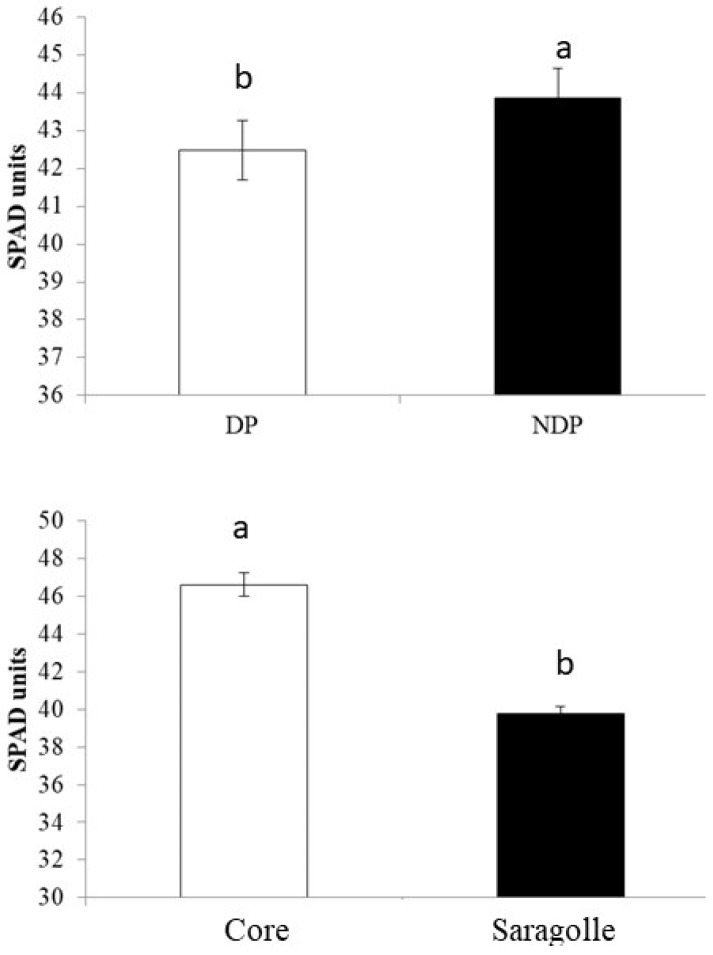
Main effect of clipping management (**top**) and variety (**bottom**) on SPAD index. Bar plots of the means overlaid by standard error bars. Lower letters above the columns indicate significant differences between values (*p*-value < 0.05).

The final number of spikes m^−2^ was significantly lower in Saragolle (266 spikes m^−2^) than in Core (*p*-value = 0.0018). The higher number of spikes m^−2^ for Core is attributable to the higher ratio of fertile to unfertile culms (90%) compared to Saragolle (70%). SPAD was measured on three dates (4, 13, and 20 May), SPAD did not vary significantly between dates but was consistently higher in Core (*p*-value < 0.001) (Figure 3 bottom). There was also a minor but significant effect of clipping that reduced leaf chlorophyll content slightly (−3% in clipped plots (*p*-value < 0.05). Average values are reported in Figure 3 (top).

### 2.3. Stomatal Conductance and Soil Moisture during the Reproductive Stage

Stomatal conductance (g_s_) mmol m^−2^ s^−1^ was measured weekly from 4 to 24 May, which corresponded to the phenological stages from heading to the beginning of grain filling in Core and from the end of booting to the end of anthesis for Saragolle. There was large variability in the data, especially around flowering. g_s_ was positively correlated with temperature (r = 0.81, *p*-value < 0.05). In order to account for the effect of temperature, the latter was included as a continuous covariate in the analysis of variance fitted through a mixed effect model. The random effect term was specified as a nested random effect (variety nested within blocks, nested within dates) The fixed effects terms significance (as main factors or in interaction) are reported in Table 2.

**Figure 4:**
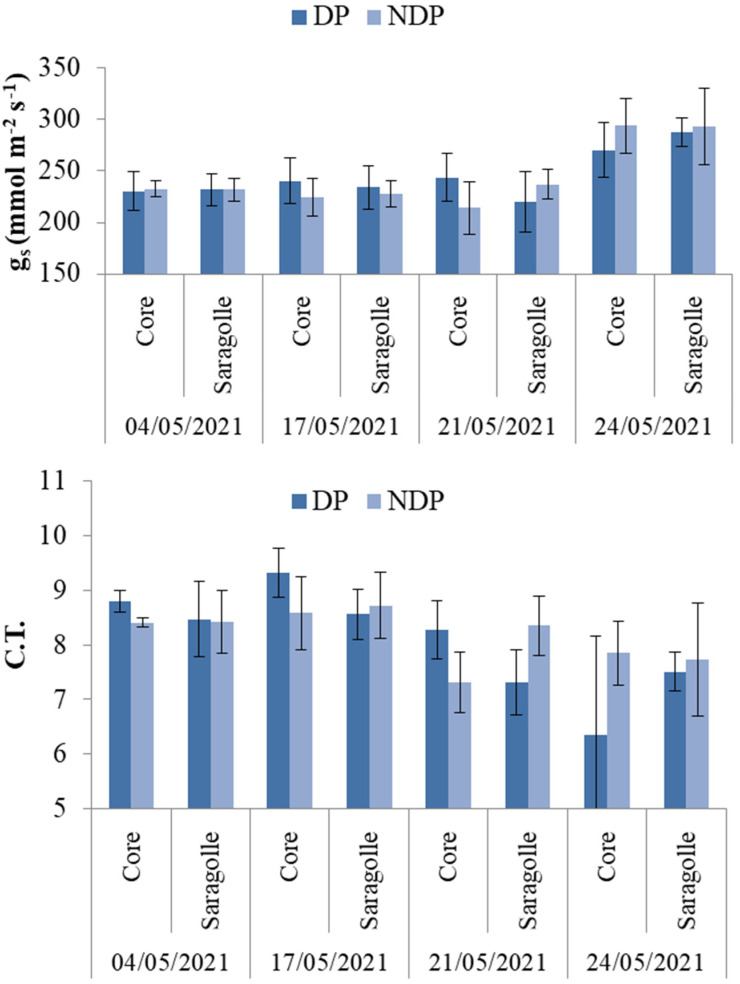
Bar plots of the mean values overlaid by standard deviation bars of stomatal conductance (g_s_) (mmol m^−2^ s^−1^) (**top**) and the ratio between g_s_ and temperature (C.T.) (**bottom**) at different dates during the reproductive stage in clipped (DP) (dark blue columns) and unclipped (NDP) (light blue columns) treatments in Core and Saragolle varieties.

Temperature was a highly significant factor, g_s_ increased around anthesis with rising temperatures (Figure 4 top). Detrending the data from the effect of temperature revealed a weak interaction between variety and clipping (*p*-value = 0.07). Figure 4 (Figure 4 bottom) displays the ratio between g_s_ and air temperature across varieties, clipping regimes, and dates. This new variable (C.T), which can be viewed as g_s_ standardized for temperature, shows that stomatal conductivity decreases toward flowering with an opposite trend in Core and Saragolle, the modern variety transpiring slightly more under DP and Saragolle, which show no variation due to clipping.

g_s_ never decreased below 200 mmol m^−2^ s^−1^ indicating that stomatal closure never occurred during the survey (even during hot days with temperatures above the average of the period). Soil water monitoring was carried out from April to June, a period corresponding to the phenological transition from booting to grain filling.

**Figure 5:**
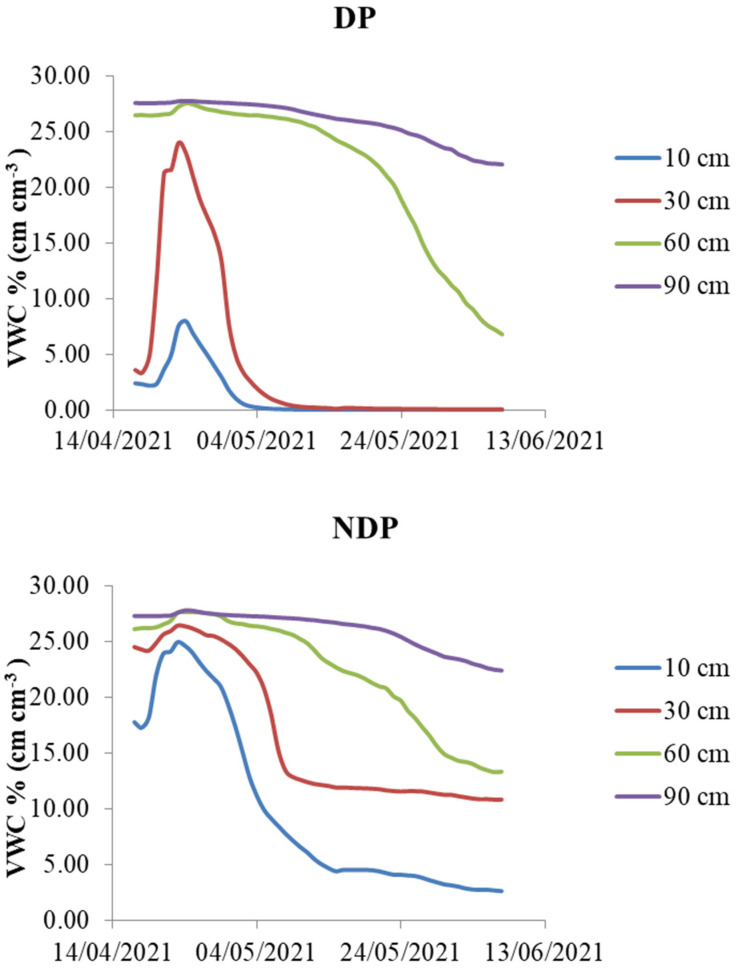
Volumetric water content % (VWC cm cm^−3^) during the reproductive stage under dual-purpose (DP) and conventional (NDP) management at the four soil depths where the probes were installed (10 cm, 30 cm, 60 cm, 90 cm).

Inference on water depletion was only carried out for Core due to the lack of replicates for Saragolla. Soil water depletion curves during the reproductive stage are depicted in Figure 5.

**Figure 6:**
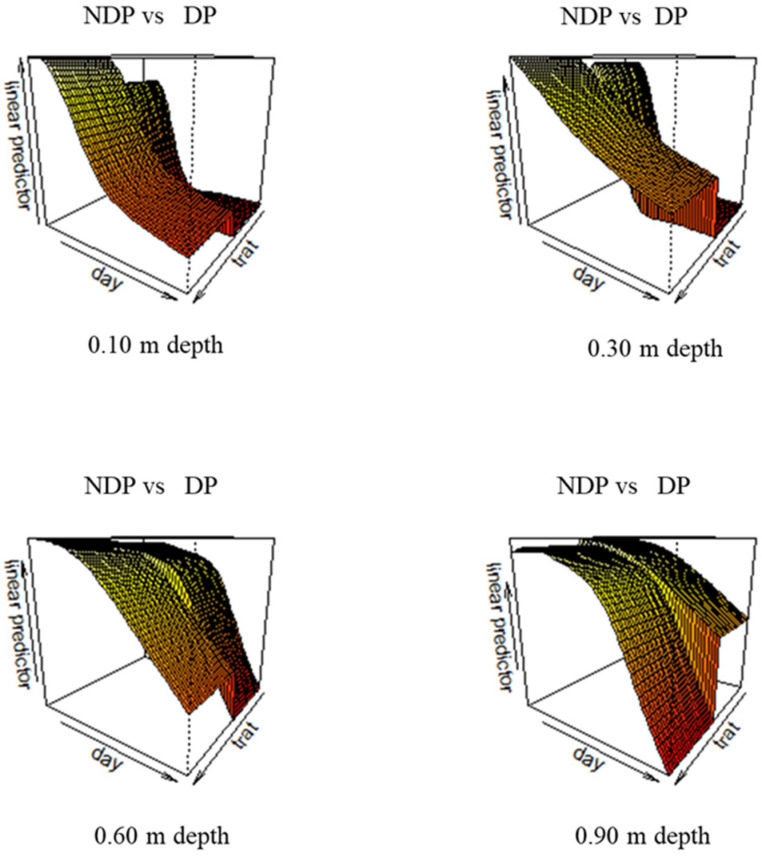
Gam model trend surface at the four soil depths: 0.10 m; 0.30 m **top left and right**; 0.60 m and 0.90 m **bottom left and right**. The three-dimensional graphs show the model predicted values of volumetric water content: *y*-axis «linear predictor»), for the two clipping regimes: *z*-axis «trat» for unclipped (NDP) (foreground) and dual purpose (DP) (background) during the reproductive stage (*x*-axis «day»). Records from 17 April–7 June 2021).

Results of the generalized additive models (GAM) for water depletion patterns in clipped and unclipped Cores are shown in Table 3. The GAM models’ predicted pattern of water depletion during the reproductive stage in clipped (DP) and unclipped plots (NDP) for the four soil layers is depicted in Figure 6.

In surface soil (10 cm) at the beginning of the measurement period, the model predicts more water available in unclipped plots, while the difference between treatments is almost negligible at 0.6 m depth (Figure 6 bottom left), and the pattern is inverted in the deepest layer (0.9 m, Figure 6 bottom right), where more water is available under dual-purpose. The percentage of explained variation increases from 46% to 88% from surface to deep soil, indicating more accurate model estimates in deep soil. By anthesis under DP, surface soil water was depleted at 0.6 and 0.9 m depth; volumetric water content was 8 and 24 g cm^−3^, respectively, equivalent to 23% and 73% of field capacity.

Under NDP, surface soil was around 37% of field capacity, while more intense dewatering occurred in deeper soil; at maturity, water content was around 41% and 62% of field capacity at 0.6 and 0.9 m depth, respectively. Under both treatments, at the end of the cycle, substantial water was still available at 0.9 m, which is above the root front (roots were found at 1.20 m depth; see Section 2.4).

As mentioned, Saragolle moisture data are not replicated, so no inference is possible, but consistently, in large Core plots by anthesis, large water reserves were still available between 0.6 and 0.9 m; during grain filling, soil water content was about 62% and 68% of FC in clipped and unclipped plots, respectively.

### 2.4. Root Biometry

Root traits were affected by clipping, showing for some traits a significant interaction between clipping and variety (see Table 4).

**Figure 7:**
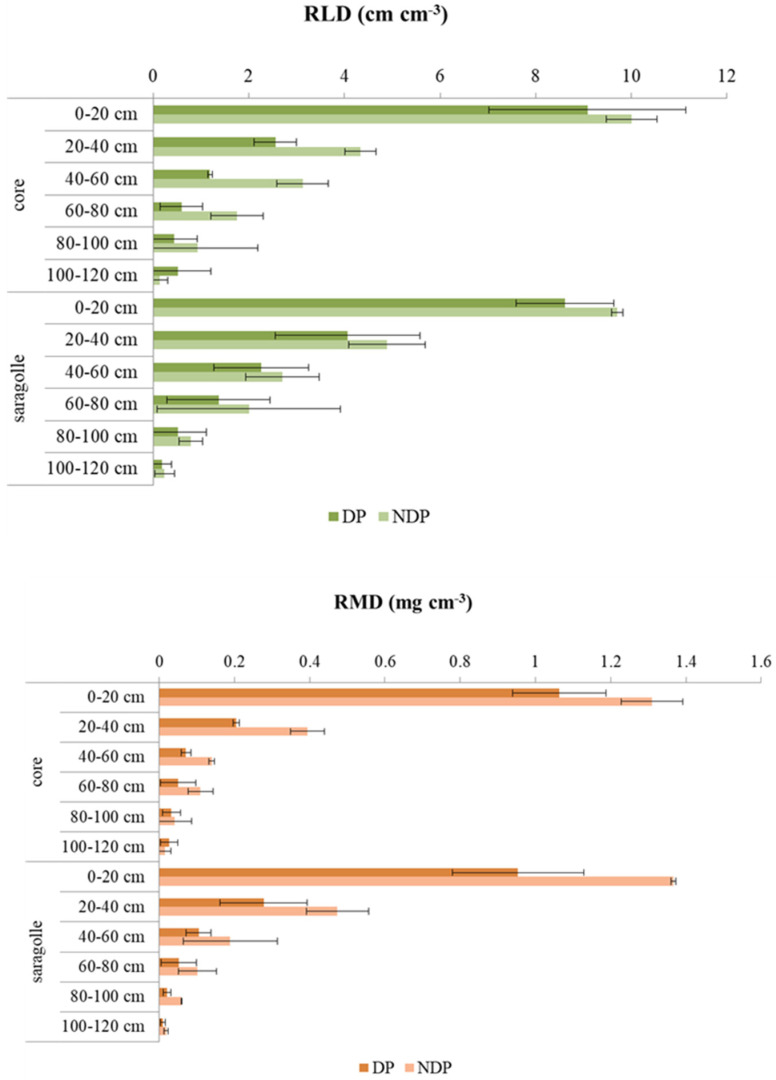
Root length density (RLD) (**top**) and root mass density (RMD) (**bottom**) in Core and Saragolle varieties under dual-purpose (DP) and conventional management (NDP) of six soil layers from surface soil to 1.20 m. Means are overlaid by standard deviation bars.

Root length and mass density average values per soil strata are shown in Figure 7.

Root length density (RLD) (Figure 7 top) was reduced by clipping, especially in Core and much less in Saragolla. The reduction in Core was evident between 0.20 and 0.60 m (Figure 7 top). DP reduced RLD by 57% in Core and by 22% in Saragolle. The differences between cultivars, however, were not significant. Topsoil was densely colonized under both clipping regimes and by both varieties (averages of 9–10 cm^−3^ in clipped and unclipped plots). No significant variation among cultivars or clipping regimes could be found in deep soil, where root density was very low and highly variable: the variation coefficient (CV%) of RLD in the soil strata 0.6–1.2 m ranged from 98% to 108% in Core for NDP and DP, respectively, and between 72% (NDP) and 104% for Saragolle (DP).

**Figure 8:**
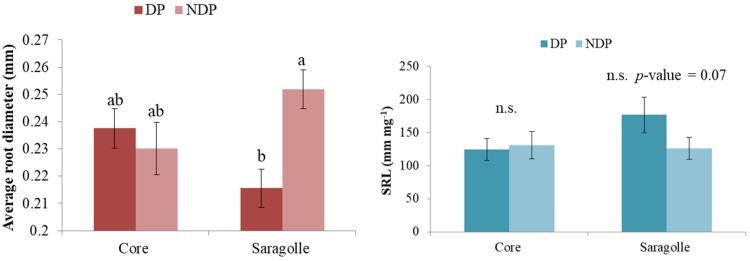
**Left**: interaction of wheat variety and clipping management on root average diameter, bar plots of the means overlaid by standard error bars; different lowercase letters over the bars depict significant differences between means (*p*-value < 0.05). **Right**: interaction of wheat variety and clipping management on specific root length (SRL), bar plots of the means overlaid by standard errors bars with indication of significance (n.s.= non significant).

RMD was even more sensitive to clipping and showed a significant depth by treatment interaction DP markedly reduced root mass in the first 0.6 m of soil. RMD values did not differ significantly between varieties. RMD was linearly correlated to RLD (RMD = 0.1259RLD − 0.082; R^2^ = 0.93) (without differences between species or treatments, *p*-value < 0.05). both RMD and RLD decrease with depth following the typical sigmoidal shape found in the literature. As mentioned in the full model, the variety effect was not significant, but when the ANOVA was computed on single varieties, RMD showed a significant reduction under DP in both Core and Saragolle (*p*-value < 0.001), and in Core but not in Saragolle, there was also a significant depth x clipping interaction (*p*-value = 0.02). For RLD, clipping was highly significant for Core (*p*-value = 0.007) and less for Saragolle (*p*-value = 0.03), depth was also highly significant (*p*-value < 0.001) but there was no interaction between clipping and depth. For both varieties, clipping and depth explain 99% of the variability in the data (squared correlation between observed and predicted values). Root average diameter showed a significant interaction between treatment and variety, with the largest diameter found in NDP Saragolle, values significantly different from DP Saragolla (*p*-value < 0.05), and no variation with depth (Figure 8 left). Specific root length (SRL mm mg^−1^) which was negatively correlated to average diameter (r = −0.83, *p*-value < 0.05) showed a tendency for interaction between clipping and variety (*p*-value = 0.05) with Saragolle increasing SRL under dual purpose (Figure 8 right).

## 3. Discussion

### 3.1. Fodder Biomass, Grain Yield and Weather Pattern

Our experiment shows no variety differences in fodder biomass, and no yield penalties associated with dual-purpose use. Our values of fodder biomass are lower than those reported in the literature, e.g., values for early-sown winter cereals at the onset of the grazing period in high rainfall areas of Australia, which range from 1 to 2.5 t DM/ha [30], and those of Francia et al. [31] for oat (1 t DM/ha) and barley (2.2 t DM/ha). They are also lower than the 0.9 t DM/ha of early-sown Triticale [26]. This can be ascribed to our late sowing, followed by a period of cool temperatures, that slowed growth. Sprague et al. [30] tested the effect of sowing date on forage availability and found that a 1-month delay in sowing reduces biomass by 63% and that for late sowings even a 1-week delay reduces biomass to 1/3. Giunta et al. [26] reported wide year-to-year variability in forage production for the old winter wheat cv Cappelli (from 0.80 to 3.28 t DM/ha). Strong inter-annual variability in forage yield was also reported by [19] for barley in Spain (0.8–3.6 t DM/ha) but for a growing period of 100 days. Early sowing, from 2 to 4 weeks earlier than grain-only crops, is recommended [32], and hence a long growing season is required to maximize both forage and grain production. At our latitudes, however, early sowing is seldom possible due to a late onset of autumn rain; most commonly, winter crops are sown from the beginning of November to the end of November. A simulation study undertaken in Australia’s high rainfall zone demonstrated that in areas with Mediterranean climates, early sowing windows for winter cereals occur with a quite low frequency (<30% of years) [33]. To facilitate the adoption of dual-purpose management, a feasibility study based on climatic data time series and forecasts in southern Italy would be valuable. Our weather data, for instance, show that in this particular year, the onset of fall rain was delayed until mid-November, thus early sowing could not be carried out without irrigation.

The overall trend of total shoot recovery after clipping indicates that both cultivars tested in our study possess a certain degree of grazing tolerance. As for grain production, the yield was not significantly different, although Core outperformed Saragolle in both clipped and unclipped plots. The lack of significant variety effect can also be attributed to the experimental design which allows estimating the main factor effect with less accuracy. A higher yield of modern wheat is in agreement with literature reporting a lower yield potential of old cultivars [34,35]. Dual-purpose management did not penalize yield, and only a slight, non-significant positive difference in favor of unclipped plots was found for Saragolla. A yield penalty of up to −18% was reported by [26] for tall winter wheat cv Cappelli, and the authors explain how late clipping impacted yield by reducing the number of spikes m^−1^ from 312 to 260 without affecting spike fertility. Similar yield reductions in clipped plants were reported for semi-dwarf cultivars of wheat and triticale [18,36]. An average penalty of 14% was reported for spring wheat, and the yield penalty can be reduced by a combination of low grazing intensity and early sowing [37]. More than 270 yield data from dual-purpose trials (either based on simulated or real grazing) showed that yield differences between clipped and unclipped plots can be negative or positive displaying a very large range (from −36% to +75%) with an average of 7% yield loss [32]. Clipping increased yield whenever it was associated with reduced water use before anthesis. Our lack of significant yield penalties is possibly due to the early defoliation and the low defoliation intensity (the crop was clipped at 5 cm height), which allowed the re-establishment of a sufficient leaf area by the reproductive stage (see the section on phenology). Defoliation height is a very important factor. Butchcee and Edwards [10] found that clipping at 12 cm did not impact grain yield, while a 3 cm cutting reduced grain yield regardless of different varietal growth habits, and across different locations. One early (pre-joint) defoliation event did not depress grain yield in Triticale [38]. Besides the low defoliation intensity, a lack of yield differences in our trial could be due to the fact that all plants could rely on a large subsoil water availability at anthesis (see Section 2.3) plus extra rainwater at the onset of grain filling. The literature on dual-purpose indicates that yield differences (e.g., yield penalties) are strongly influenced by rain patterns from clipping to grain filling. The weather data in our case were those of an average year in terms of total rain availability; wheat grew mostly on stored water as precipitation was concentrated in the winter months. We had a drier spring compared to the long-term average (about 50% of an average wet spring); substantial summer rain in June (106 mm vs 42.1 average) 98% of which concentrated in the second week of June, that during the beginning grain filling, may have contributed to reducing differences between treatments.

### 3.2. Phenology and SPAD Index

Clipping slightly delayed phenology in both cultivars. A delay in phenology due to defoliation has been reported [32]. Tian et al. [36] found that early clipping in wet years minimally impacted wheat phenology (<3 days at anthesis and 5 days at maturity) but reduced the number of spikes per square meter and reduced grain yield by 18%; in our case, the final number of spikes was unaffected. Clipping delayed the rate of leaf emergence in triticale and delayed anthesis by 2–7 days across several genotype x environment combinations [16]. Clipping delayed phenology (the time of booting, heading, anthesis, and maturity) with a significant interaction between cutting, cultivars, and planting dates, with the delay exacerbated by early sowing [12]. The impact of clipping on phenology also depends on the interaction with drought stress, which also impacts plant development. The post-grazing growth rate, however, was significantly lower or higher in grazed plots in dry and wet years, respectively [11]. Drought stress impacts phenology [39]. The delay in phenology did not penalize yield in our data; this could also be due to the fact that after clipping under both treatments, both cultivars could rely on a substantial amount of stored water (see Section 2.3).

Leaf chlorophyll content as indirectly measured in SPAD units was reduced by clipping, and this is not in agreement with [40] who report how even intense biomass removal did not change leaf chlorophyll. Li et al. [41] showed seasonal trends and date-to-date variation in the chlorophyll content of wheat and rice and correlated it to carboxylation parameters of photosynthesis. SPAD was consistently lower for Saragolle, which suggests a lower nitrogen use efficiency for this cultivar. Under organic farming in southern Italy, all durum wheat modern varieties outperformed old varieties Cappelli and Saragolla showing a higher nitrogen use efficiency and more responsiveness to leaf fertilization [42]. Bochicchio et al. [43] reported a lower chlorophyll content index in Saragolle lucana, the same variety as in our experiment, compared to modern varieties.

Despite the lower leaf nitrogen content, durum grain protein content tends to be higher for old cultivars, showing a negative correlation with the year of release, possibly due to a dilution effect [44]. The protein content of grain and Semolina has also been shown to be higher for old Sicilian bread wheat landraces compared to modern varieties [45]. Giunta et al. [46] reported that stems also contribute to protein translocation. In our case, stem length at harvest was significantly higher for Saragolle (107 vs 54 cm from the base of the stem to the node of the flag leaf, respectively (*p*-value < 0.001), and this may have counterbalanced the lower leaf nitrogen content.

### 3.3. Stomatal Conductance and Soil Water during the Reproductive Stage

Stomatal conductance did not differ between cultivars and was not significantly impacted by clipping. The transpiration rate increased after flowering, and this increased water demand was correlated to air temperature. If the effect of temperature is discounted, however, g_s_ tends to be higher before anthesis, a period during which soil water is very large in both clipped and unclipped plots. No significant effect of treatment was detected; there was only a trend in the data indicating that the modern variety at most dates tends to transpire more in clipped plots, while Saragolle shows no effect and keeps up the same g_s_ of the modern wheat. A higher transpiration rate due to clipping was found at early sowing dates for one variety, but in the same trial, for most cultivars, the transpiration rate was unaffected by clipping [40]. Based on genetic background, we would have expected differences between modern and old wheat, but Saragolle only showed a minimal, non-significant positive difference in stomatal conductance. In the literature, it is frequently reported that stomatal conductance is higher for modern wheat compared to old varieties. Le Cain et al. [45] reported that modern varieties possess a higher stomatal density than tall winter wheat. Fischer et al. [46] compared a set of irrigated semi-dwarf bread wheat varieties released between 1962 and 1988 and found that stomatal conductance was positively associated with the year of release, with the newest varieties showing higher g_s_, and also higher yields. This is also documented by Roche [47], who argued that breeding for higher yields inadvertently selected for higher g_s_; this co-variation of yield and g_s_ was found in spring wheat [48] and durum wheat [35], but also in dicots such as soybean [49] and cotton [48]. Giunta et al. [50] showed that stomatal resistance is negatively associated with cultivar year of release and is generally higher in tall varieties (including the old cultivar Saragolla) compared to modern semi-dwarf cultivars. Tall varieties tend to have cooler canopies due to their lower aerodynamic resistance, a trait that greatly affects transpiration but goes in the opposite direction of leaf resistance. Transpiration is also controlled by water uptake capacity, which is in turn regulated by soil water availability and root acquisition efficiency. Nakhforoosh et al. [51] found that most wheat landraces are water-spenders compared to moderns. Regardless of the perspective, anatomical differences in favor of Core (higher stomatal density reported for modern wheat) Saragolle kept up with high transpiration rates regardless of defoliation; this suggests a compensatory mechanism either due to an effective canopy rebuilding capacity or sustained water uptake under both clipping regimes. These hypotheses remain to be validated. Regarding the effect of clipping on soil water depletion, our partial data analysis for the modern Core indicates that clipping was significant both as a fixed factor and as a smoothing term, meaning that not only was there a significant difference in average water depletion in all soil layers (a parametric term) but there was also a difference in the temporal dynamics of depletion (a significant difference between smoothing terms).

As shown in Table 3, the effective degrees of freedom (edf) estimated from generalized additive models, which are always indicative of a moderate to high degree of non-linearity, are always different between clipped and unclipped plots; the edf indicates a higher degree of non-linearity under DP, especially in topsoil, and this is due to the fast dewatering followed by an asymptotic decline (between 0.10 and 0.30); under NDP, the edf are lower and the pattern is almost linear, indicating a more constant water withdrawal.

Model accuracy increases with depth; the poor predictive ability in topsoil can be due to surface heterogeneities caused by the erratic distribution of soil cracks: A more intense soil cracking occurred in defoliated plots (personal observation) which may be at the origin of the rapid water loss consistent with the rapid rise of air temperature in April. By anthesis, less water was available in surface soil, and depletion was more intense in intermediate layers (0.60 m), as the drying topsoil may have forced the plant to extract more water from beneath. The possible role of dual-purpose as a pre-anthesis water-saving agronomic strategy has been one of the main points of strength in favor of the adoption of this technique. While necessarily less water is transpired by defoliated plots, at least soon after clipping due to the removal of leaf area, it is not guaranteed that this spared water can be used later (e.g., during the critical stage of anthesis) or rather it is lost through evaporation/deep drainage. Our data suggest that under DP—at least in years characterized by a post-clipping dry spell—surface soil water is lost before anthesis; this might be partially due to a more intense transpiration rate of the rebuilding canopy (higher transpiration rates normalized by temperature) or to bare soil evaporation. Based on porometer data, we hypothesize that water is mostly lost through evaporation, as differences in stomatal conductivity between clipped and unclipped are very low and non-significant. Kelman and Dove [11] found that in years characterized by no rain after clipping, more water was depleted between 0.6 and 1.70 in grazed plots compared to ungrazed controls. Virgona et al. [18] found that defoliation reduced global water use efficiency by 18% due to sustained water use throughout grain filling. Deferred water use was observed by these authors: grazing reduced water uptake during and soon after grazing; at flowering, though, grazed plants depleted soil more than ungrazed ones. Giunta et al. [26] found that in the old tall durum variety, Cappelli total water use was not affected by grazing since in defoliated plants higher evaporative losses were compensated by reduced transpiration. Therefore, while water partitioning between evaporation and transpiration was altered by clipping, total water use and transpiration efficiency did not differ. Bonachela et al. [19] found that dual purpose was beneficial in years of low rain availability in post-grazing since water use was deferred until anthesis; this positive effect of clipping on water use can be reduced in cases of high evaporative losses during the post-grazing period. Moreover, in wet years, defoliated plants benefit less than unclipped plants from any extra available water because of reductions in water uptake. The impact of clipping on subsoil water use is affected by inter-annual variability. The one-year duration of our trial certainly limits the representativeness of our result, although 2021 could be considered an average year for the area, with rain concentrated in the winter months and a relatively dry spring. Our soil water data show that the profile was fully recharged at the beginning of the dry season (soil moisture was around the field capacity), substantial water was still available in deep soil at anthesis, and further rain recharged the water reserves during grain filling. In these conditions, dual-purpose did not lead to a water reserve build-up since there were high losses from surface soil. Moreover, soil moisture data in unclipped plots suggest there was less efficient exploitation of deep water. More work is needed to assess the performances of dual-purpose wheat in years during which the soil profile has not been fully recharged and hence pre-anthesis water savings or losses may impact yield much more than in wet years. Under average years dual-purpose management supplies extra forage resources and if old tall varieties are used there is an extra supply of stubble and straw, a non-negligible gain considering that in some Mediterranean countries such as Algeria durum straw sale price can be as high as 30% of that of grain [52].

### 3.4. Root Biometry

Root biometry showed interesting features in response to dual-purpose management. In our experiment, clipping did not affect root density in deep soil, which is similar under both treatments and for both cultivars. No differences in root depth due to clipping were found by [23] on spring wheat and were also reported by [53] for barley and triticale. Root length density, though, was halved in repeatedly clipped wheat [23] and slightly reduced in barley and triticale [53]. The effect of defoliation on roots has been early studied in pasture crops, and it is generally acknowledged that root elongation rate and length density tend to be strongly reduced soon after shoot removal to prioritize carbon use for the re-establishment of the photosynthetic canopy, but differences exist between crops and between cultivars. Grazing-tolerant crested wheatgrass (*Agropyron desertorum*) showed a severe root length reduction (−50%) under clipping compared to grazing-sensitive blue bunch wheatgrass (A. spicatum), which was unaffected but later suffered high long-term root mortality possibly due to insufficient shoot recovery [54]. Defoliation sharply reduced perennial ryegrass root elongation soon after clipping, followed by a root growth recovery stage [55]. Root elongation rate fell sharply a few days after clipping in several forage grasses and legumes: perennial ryegrass, timothy cocksfoot, and red clover; white clover, however, did not cease to grow roots, and overall, the reduction was generally proportional to the defoliation intensity for grasses but not for legumes [56]. Root extension and branching were also reduced soon after defoliation in wallaby grass (*Danthonia caespitosa*), old man saltbush (*Atriplex nummularia*), bladder saltbush (*A. vesicaria*), and lucerne (*Medicago sativa*) [21]. These variations in root growth impact nutrient foraging capacity as well as water acquisition. In [57], interesting data were reported on root N-uptake and re-translocation after shoot removal, indicative of a compensatory mechanism: nitrogen acquisition efficiency (nitrogen uptake per unit root weight) increased with defoliation for several grasses (e.g., *Agrostis castellana*, *Festuca rubra*, *Lolium perenne,* and *Poa trivialis*). Nitrogen uptake on a plant basis was reduced in *Lolium* and *Poa*. In our experiment, defoliation drastically reduced both root mass and length density in Core between 0.30 and 0.60 m, and this was consistent with slower and lower dewatering below in this strata. Since all shoot mass was recovered after clipping, the disinvestment in roots can be considered a grazing-tolerant mechanism to redirect carbon to canopy rebuilding. Saragolle showed an interesting root response to clipping: it marginally reduced root length density but reduced the metabolic cost of roots by decreasing root mass, increasing SRL, and reducing root diameter under clipping. This means that Saragolle was able to reestablish aboveground biomass (possibly redirecting carbohydrates to the shoot), but this was not performed at the expense of soil exploration. In unclipped plots, Saragolle produced thicker roots, and soil water data provide a first indication of more intense dewatering at all strata above 60 cm compared to clipped plants; this might be due to a greater density of nodal roots, which have a larger diameter, larger water uptake per unit length, and higher hydraulic conductivity than seminal roots [58,59]. Defoliation in fact has been reported to reduce nodal roots [23] which are also acknowledged for a higher nutrient acquisition efficiency [60]. Core, however, shows no significant variation in diameter between treatments despite the greater reduction in root mass and length density under clipping, so there is no evidence of predominant clipping incidence on nodal roots. The difference in root morphology between cultivars can be due to the different phenology: clipping may have impacted nodal root development only in Saragolle, which showed a delayed phenology, but Saragolle reduces more root mass than root length, indeed showing a tendency for increased SRL and increased root surface area. An intriguing hypothesis is that Saragolle is capable of producing thicker roots (possibly capable of higher water uptake rates) if C is not limiting and switches to thinner, low-cost roots if building materials are lacking. In fact, a large diameter is a proxy for a larger xylem vessel diameter [61]. If this is true for Saragolle, it would imply a different hydraulic conductivity, with consequences on water use efficiency. Root anatomical plasticity is a valuable asset under limited resource availability. Compared to Core, under DP, Saragolle reduced the metabolic cost of root exploration (less root mass, less C used for roots) without reducing root foraging capacity (an increased length-to-mass ratio).

## 4. Conclusions

Our work aimed at testing the dual-purpose attitude of two durum wheat varieties: a modern cultivar and an ancient tall variety, in a region of medium-high winter rainfall water availability in southern Italy.

Our results show that:

- One early defoliation event did not penalize grain yield. Both modern and ancient wheat fully recovered canopy losses. Defoliation delayed phenology but had no consequences on the final number of spikes per square meter.
- The two varieties showed differences in leaf chlorophyll content, which was marginally affected by clipping. Stomatal conductivity did not differ between varieties; both maintained high transpiration rates throughout the reproductive season.
- Our partial data (only available for modern wheat) show that soil water depletion was influenced by clipping. Defoliation did not result in a reduction in pre-anthesis water reserves, possibly due to a compensation of lower plant water demand with higher evaporative losses and delayed phenology.
- Dual-purpose management markedly affected the root sync, and root mass was severely reduced by clipping. Defoliation also reduced root length, but this was more drastic for modern wheat. Saragolle lucana variety when defoliated shifted to thinner roots so root length was less impacted than root mass: a mechanism that can contribute to reducing the metabolic cost of root exploration without compromising root foraging capacity.

Extension of results for management implications requires multi-year studies in Mediterranean environments or feasibility studies based on weather data time series or simulation. Data from this experiment, though, provide a preliminary management indication that in an average year for the area (of medium–high winter rainfall availability), dual-purpose management is feasible for durum; if ancient tall varieties are used, extra stubble would be available for livestock enterprises.

Our results can also be useful for suggesting mechanisms and parameters for specific components, e.g., crop models aimed at crop responses to dual-purpose techniques. In particular, our root results point to belowground plasticity as a trait worth focusing on due to the many implications of plant–soil relations, ranging from resource acquisition capacity to ecosystem services associated with root biometry.

Future work may address unexplored agronomic management issues for the area, such as multiple sowing dates, clipping events, and defoliation intensity. Our findings on roots raise novel hypotheses regarding possible anatomical differences between modern and ancient wheat varieties, which are relevant in the context of root hydraulics. More work on root phenotyping may help define a root ideotype to be paralleled with the shoot ideotype, especially suitable for dual-purpose systems.

## 5. Materials and Method

### 5.1. Experimental Setup

Experiments were conducted in Bella (PZ), Basilicata region, southern Italy (40°42′ N, 15°32′ E) on a silty clay loam soil (20% sand, 35% clay, 45% silt) in 2021. The experimental design consisted of a split-plot design replicated six times. Two varieties of durum wheat (*Triticum durum* Desf.), the modern cv Core and the old tall cv Saragolle lucana, were assigned to the main plot (2 × 2 m, 12 plots), and two clipping regimes, conventional (NDP) and dual-purpose (DP) management, were assigned to the sub-plots (1 × 2, 24 plots). The crop was sown on 15 November 2020, at a density of 450 plants m^−2^ and a distance of 17 cm between rows for both cultivars. The DP treatment consisted of simulating grazing by clipping plants at 5 cm from the ground approximately 3 months after sowing (15 February 2021) at the end of the tillering stage, visually checking that the crop had not yet passed the first hollow stem phenological stage. Nitrogen fertilization was applied at low dose (100 kg N hectare^−1^) 35% application during the tillering stage (20% at 4 leaves) and the remaining 80% was applied during the shooting stage. Nitrogen was supplied in the form of ammonium nitrate. This low fertilization rate was chosen in order to avoid lodging of the tall variety.

### 5.2. Fodder Biomass, Grain Yield and Weather Pattern

After clipping fodder biomass was weighed before and after oven-drying at 70 °C to measure fresh and dry matter fodder yield. Plant biomass was resampled on 4 May to measure canopy recovering capacity. On 20 May 2021, crop height (from the base of the stem to the flag-leaf node), number of fertile spikes, and number of unfertile culms were measured. The crop was manually harvested on 6 July 2021, and grain was threshed in August with a laboratory thresher (Wintersteiger LD 180); at the time of threshing, the seed humidity level was 11%. Meteorological data (minimum and maximum daily temperatures, daily cumulative rain) were retrieved from the closest meteorological weather station of Bella S. Antonio Casalino (downloaded from http://centrofunzionalebasilicata.it, accessed on 1 September 2022).

### 5.3. Phenology and SPAD Index

Phenology was recorded on four dates, visually scoring the phenological stage on a sub-plot basis. The SPAD index was measured on the flag leaf with a leaf transmittance leaf clip chlorophyll concentration meter (MC-100 Apogee Instruments, Logan, UT, USA). Measurements were carried out on three dates during the reproductive stage (4, 13, and 20 May), each time SPAD values were calculated by averaging readings from three plants per sub-plot plant.

### 5.4. Stomatal Conductance and Soil Water during the Reproductive Stage

Stomatal conductance was measured on fully developed and intact leaves with a handheld steady-state porometer (SC-1, METER Group, Pullman, WA, USA). Measurements were carried out on four dates during the reproductive stage: 4, 17, 21, and 24 May 2021. Stomatal conductance was measured on three out of six replicates due to the necessity of sampling plants within a narrow time window. Measurements were taken during the central hours of the day (between 10:00 a.m. and 1:00 p.m.) under a clear sky. We measured a minimum of three leaves (from three different plants) per sub-plot; up to five records were taken in case of high within-plot variability. Air temperature records were also available for each measure.

Soil water content was measured during the reproductive stage (from 17 April 2021 to 6 June 2021) by installing capacitive moisture probes (Vegetronix VH400). Probes were installed in two replicates of the modern wheat Core and in one replicate of Saragolle. For each sub-plot, four probes were installed in the inter-row at the following soil depths: 0.10, 0.30, 0.60, and 0.90 m. Probes were cabled to a data logger (LOGGER8-USB: 8 channel USB sensor reader) that was programmed to take four readings per day (6 h intervals between readings). Sensor data in volts were converted into volumetric water content (VWC %) using the calibration equation for clay soil developed by Bitella et al. [62].

### 5.5. Root Biometry

Roots were sampled on 19 June 2021. Roots samples were taken by soil coring using a hydraulic penetrometer and soil drilling machine (Penetrometro TG 63—100 Pagani, Italy) equipped with still coring tubes filled with extractable PVC tubes (inner diameter 8 cm, 60 cm height). The coring tube was centered on the row directly on the plant (which was previously clipped to the ground), and cores were taken at two consecutive depths (0–0.60 m and 0.60–1.20 m). Root samples were taken in two out of six replicates, collecting two sub-replicates per sub-plot; sub-replicate values were averaged for the analysis. Immediately after sampling, soil cores were stored at −20 °C for a few weeks. The frozen cores were then subdivided into 20 cm increments with the aid of a circular saw. Sub-cores were unfrozen overnight in water and sodium hexametaphosphate (15 g/L) and were then elutriated over stacked soil sieves from 2 mm to 0.4 mm). Root fragments were stored in ethanol (50% *V*/*V*) at 4 °C, scanned, and subjected to image analysis using WinRhizo Arabidopsis V2009c image analysis software (Regent Instruments Inc., Quebec, Canada). After scanning, roots were oven dried at 70 °C for dry biomass quantification. The following root parameters were quantified and analyzed:

- Root length density (cm cm^−3^) (RLD);
- Root volume (cm^3^ cm^−3^);
- Root average diameter (mm);
- Root mass density (g cm^−3^) (RMD);
- Specific root length (mm mg^−1^) (SRL).

### 5.6. Statistical Analysis

The effects of variety and clipping were tested alone or in interaction through the analysis of variance. The split-plot experiment required an analysis of variance, which specifically includes nested error terms to account for the hierarchic structures of the experiment. The model specification was performed within the framework of mixed effects models, which included both fixed effects and nested random error terms. The random effects terms (variety nested within blocks), as nested random effects, allow for different intercept at the level of variety within blocks, while fixed effect terms are required to model the systematic effect of a given factor [63]. Random terms were also specified in cases of repeated measurements (different soil depths) or longitudinal data (different dates of measurements) and included in the nested error structure. Mixed modeling was carried out using the R package; lme4 *p*-values were computed through Satterthwaite’s method [63]. ANCOVA model was specified for the analysis of stomatal conductance using air T °C as a continuous variable fixed effect.

Soil moisture data from clipped and unclipped plots were analyzed within the framework of generalized additive models (GAM) [64] to model the non-linearity of soil moisture time series. GAM’s specification includes both parametric terms and a smoothing term. The smoothing term allowed for modeling the temporal pattern of soil water depletion in clipped and unclipped plots. Models were computed for the four soil water time series at each location (0.10, 0.30, 0.60, and 0.90 m soil depths). The GAMs were fitted by penalized likelihood maximization, and smoothing parameters were automatically chosen to minimize the generalized cross-validation criterion. GAM computation and goodness-of-fit assessment were carried out using the R package “gam” [65].

## Figures and Tables

**Table 1:** Grain yield (t ha^−1^) cultivars average values and standard deviation for clipped (DP) and unclipped (NDP) plots.

	Yield (Tons ha^−1^)			
	DP		NDP	
Variety	Mean	St.dev	Mean	St.dev
Core	2.95	1.00	3.22	0.63
Saragolla	2.43	0.59	2.47	0.41

**Table 2:** Type III analysis of variance table with Satterthwaite’s method. Significant terms (*p*-value < 0.05) are reported in bold.

Source of Variation	Pr(>F)
T °C	**0.0002**
Date	**0.0521**
Variety	0.5476
Clipping	0.9897
Date × variety	0.9507
Date × clipping	0.4755
Variety × clipping	0.0702
Date × Variety × clipping	0.1433

**Table 3:** GAM models summary statistics, from left to right: depth of the moisture probes, parametric terms, coefficient estimates and relative *p*-values, smooth terms, effective degrees of freedom (edf), determination coefficient, and generalized cross validation score (GCV).

	Parametric Coefficients			Approximate Significance of Smooth Terms:			Goodness of Fit	
Soil Depth (m)	Parametric Term	Estimate	Pr(>|t|)	Smooth Term	edf	*p*-Value	R^2^ Adj	GCV
0.1	Intercept	3.428	**<0.001**	s(day) DP	5.382	**<0.0001**	0.44	36.852
	clipping—NDP	4.242	**<0.001**	s(day)NDP	3.645	**<0.0001**		
0.3	Intercept	6.301	**<0.001**	s(day) DP	5.607	**<0.0001**	0.48	68.139
	clipping—NDP	8.010	**<0.001**	s(day)NDP	1.688	**<0.0002**		
0.6	Intercept	21.630	**<0.001**	s(day) DP	5.126	**<0.0001**	0.79	9.322
	clipping—NDP	0.711	**<0.001**	s(day)NDP	2.785	**<0.0001**		
0.9	Intercept	26.410	**<0.0001**	s(day) DP	2.681	**<0.0001**	0.874	0.55
	clipping—NDP	−0.933	**<0.001**	s(day)NDP	5.204	**<0.0001**		

**Table 4:** Analysis of variance for root traits, *p*-values < 0.05 are reported in bold. Asterisk depicts factors significance (significance codes: <0.001 ‘***’ 0.01 ‘**’).

	Source of Variation	Pr(>F)	
RLD (cm cm^−3^)	Depth	**1.59 × 10^−8^**	***
	Variety	0.506854	
	Clipping	**0.000376**	***
	Depth × variety	0.831928	
	Depth × clipping	0.140179	
	Variety × clipping	0.19326	
	Depth × variety × clipping	0.53003	
RMD (mg cm^−3^)	Depth	**2.16 × 10^−12^**	***
	Variety	0.494564	
	Clipping	**3.64 × 10^−5^**	***
	Depth × variety	0.710335	
	Depth × clipping	**0.001257**	**
	Variety × clipping	0.308827	
	Depth × variety × clipping	0.738796	
SRL (mm mg^−1^)	Variety	0.36086	
	Clipping	0.1285	
	Variety × clipping	**0.05413**	
Average diameter (mm)	Variety	0.99492	
	Clipping	0.064932	
	Variety × clipping	**0.007635**	**

## Data Availability

Data available upon request to the corresponding author.

## Aknowledgment

The authors wish to thank F. Lorenzo, technical collaborator from CREA-ZA for helping in retrieving part of the weather records.
